# Rapid Fabrication of Epidermal Paper-Based Electronic Devices Using Razor Printing

**DOI:** 10.3390/mi9090420

**Published:** 2018-08-22

**Authors:** Behnam Sadri, Debkalpa Goswami, Ramses V. Martinez

**Affiliations:** 1 School of Industrial Engineering, Purdue University, 315 N. Grant Street, West Lafayette, IN 47907, USA; bsadri@purdue.edu (B.S.); dgoswami@purdue.edu (D.G.); 2 Weldon School of Biomedical Engineering, Purdue University, 206 S. Martin Jischke Drive, West Lafayette, IN 47907, USA

**Keywords:** epidermal sensors, stretchable electronics, wireless power, hydrophobic paper, wearable stimulators, paper electronics, low-cost manufacture

## Abstract

This work describes the use of a benchtop razor printer to fabricate epidermal paper-based electronic devices (EPEDs). This fabrication technique is simple, low-cost, and compatible with scalable manufacturing processes. EPEDs are fabricated using paper substrates rendered omniphobic by their cost-effective silanization with fluoroalkyl trichlorosilanes, making them inexpensive, water-resistant, and mechanically compliant with human skin. The highly conductive inks or thin films attached to one of the sides of the omniphobic paper makes EPEDs compatible with wearable applications involving wireless power transfer. The omniphobic cellulose fibers of the EPED provide a moisture-independent mechanical reinforcement to the conductive layer. EPEDs accurately monitor physiological signals such as ECG (electrocardiogram), EMG (electromyogram), and EOG (electro-oculogram) even in high moisture environments. Additionally, EPEDs can be used for the fast mapping of temperature over the skin and to apply localized thermotherapy. Our results demonstrate the merits of EPEDs as a low-cost platform for personalized medicine applications.

## 1. Introduction

The ever-growing demand for wearable technologies capable of monitoring key physiological signals have a predicted market growth from $15b in 2015 to $150b in 2027 [1]. Wearable healthcare devices fabricated using conventional rigid platforms and silicon-based technologies have been demonstrated to be useful in the continuous collection of clinically relevant personalized information for diseases such as heart failure [2], erythema [3], and diabetes [4,5]. Unfortunately, these rigid or semi-rigid wearable devices can lead to inconsistent measurements due to their limited conformability to human skin during motion and they are often perceived by patients as uncomfortable, thus hindering their adoption.

A new class of thin, flexible, and stretchable electronics, known as epidermal electronic systems [6], has emerged as wearable healthcare tools capable of efficiently monitoring a variety of physiological signals [7] and stimulating different tissues [8]. The stretchability and low mechanical impedance (similar to human skin) make epidermal electronics suitable for continuous health monitoring using wearable devices, due to their intimate contact with the skin and their compliance to its natural moves.

Thin films of ductile metals such as gold or platinum have been extensively used in the fabrication of contact electrodes for epidermal electronics due to their chemical stability and low resistivity [9]. Several electrode designs such as serpentine patterns [10], fractal designs [11], and self-similar buckles [12] have been explored to match the mechanical impedance of human skin. The manufacture of epidermal electronics with conformable designs often require clean room processes, such as photolithography [13], wet etching [9,14], and physical vapor deposition [7] to create conductive electrodes capable of conforming to the skin. A variety of epidermal electronic systems assembled on flexible substrates have demonstrated excellent measuring performances even during stretching or severe bending, with a resolution comparable to that of advanced CMOS technologies [15]. Unfortunately, the high cost of the materials and the complexity of the fabrication processes (incompatible with large scale manufacturing) required to manufacture epidermal electronics make them unsuitable for personalized medicine applications.

Paper has become a popular substrate for flexible electronics due to its printability, low cost, light weight, and disposability [16]. Conductive inks [17,18], semiconductors [19,20], and insulators [21] can be introduced to papers to tailor the electrical properties of the final device using simple manufacturing processes such as inkjet printing [19,22], spin coating [23], or screen-printing [24] to make devices such as transistors [25], batteries [26], solar cells [27], light-emitting diodes [28], triboelectric generators [29], and antennas [30]. The limited stretchability (below 5%) of paper, however, makes paper-based electronics unsuitable for epidermal applications. Moreover, the performance of electronic devices printed on conventional paper is sensitive to relative humidity and temperature. The development of a simple method to fabricate a variety of paper-based epidermal electronic devices will be desirable to significantly reduce their cost and manufacturing time.

Several approaches have been proposed to change the wetting properties of paper to improve its electrical stability and mechanical integrity in high humidity environments [31]. Infusing hydrophobic materials such as wax or photoresists has enabled the selective modification of the wettability of the paper, enabling the fabrication of low-cost microfluidic devices [32,33]. Recently, our group introduced the selective functionalization of the cellulose fibers of paper using fluoroalkyl trichlorosilanes (R^F^) as a fast and simple way to render paper omniphobic, limiting its wettability by aqueous solutions and non-polar solvents [34]. Omniphobic paper reduces the consumption of conductive ink during printing processes, reducing the price of printed electronics, and avoids the degradation of the mechanical properties of the paper due to environmental humidity. The moisture insensitivity and light weight of omniphobic paper has promoted its use as a low-cost substrate for applications in MEMS [35], microfluidics [36], and portable analytical devices [37]. Additionally, omniphobic paper devices can be easily prototyped using a variety of scalable tools such as engravers, laser cutters, and razor printers [38].

Our previous work on paper-based microfluidics demonstrated the low-cost fabrication of analytical systems by employing a thin cutting blade [6]. Here, we demonstrate the use of razor printing to rapidly fabricate epidermal paper-based electronic devices (EPEDs). EPEDs described in this study comprise a layer of a conductive material (metallic thin film or microparticle-based ink) attached to a layer of cellulose paper rendered omniphobic through silanization. Razor printed EPEDs can be rapidly fabricated at a low cost and offer several advantages as follows: (i) They are lightweight, thin, flexible, and even more stretchable than human skin; (ii) They are capable of real-time monitoring of bio-signals such as electrocardiogram (ECG), electro-oculogram (EOG), and electromyogram (EMG) with high precision, independently of environmental moisture or sweating of the wearer; (iii) The wide variety of thin metallic films and conductive inks compatible with razor printing provides an ample range of conductive agents to tailor the functionality of the EPED; (iv) EPEDs fabricated with flexible conductive inks can be used to monitor temperature and provide localized heat therapy, and (v) The omniphobic fibers of the paper reinforce the conductive layer of the EPEDs, preserving the electrical conductivity of the device upon stretching and making these devices compatible with wireless power transfer applications.

## 2. Materials and Methods

### 2.1. Choice of Materials

We purchased Whatman#1 paper (GE Healthcare Inc., Philadelphia, PA, USA) and thin paper (70-μm-thick, Elements 300, amazon.com) to serve as substrates for the EPEDs. Thin copper foils (20-μm-thick, Kraftex Products, Gloucestershire, UK) and Ag/AgCl ink (AGCL-675, Applied Ink Solutions, Westborough, MA, USA) were employed as conductive layers. We used a solution of a long-chain fluorinated organosilane (Diisopropyl(3,3,4,4,5,5,6,6,7,7,8,8,9,9,10,10,10-heptadecafluorodecyl)silane, Sigma-Aldrich Corp., St. Louis, MO, USA) to render the paper substrates omniphobic.

### 2.2. Fabrication of EPEDs by Razor Printing

**Figure 1:**
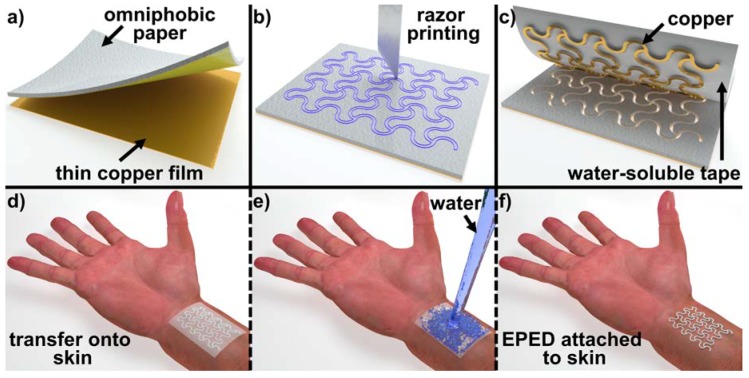
Fabrication of epidermal paper-based electronic devices (EPEDs) using razor printing: (**a**) A layer of omniphobic paper is glued to a thin metallic film that serves as a conductive layer (alternatively Ag/AgCl ink can be directly deposited on omniphobic paper); (**b**) A 100-µm-thick razor blade shapes the ensemble into a serpentine pattern; (**c**) A water-soluble tape, attached to the paper side of the EPED, is used as a temporary substrate for transfer onto skin; (**d**) The EPED is transferred onto skin previously sprayed with medical glue; (**e**) Placing the EPED under a stream of running water dissolves the temporary substrate; (**f**) EPED conformally attached to the skin.

**Figure 2:**
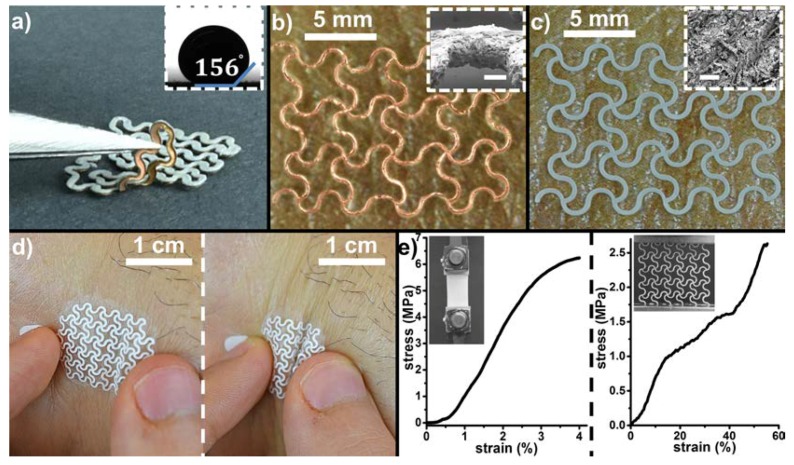
Razor printed EPEDs: (**a**) Omniphobic EPEDs comprising a thin conducting layer and a silanized paper serving as a back support. Inset shows the apparent contact angle of a 10 µL water droplet on top of the silanized paper substrate; (**b**) EPED on top of skin, with 20-µm-thick copper film as a conducting layer (copper facing up). The inset shows an SEM image of the 70-µm-thick patterned paper substrate (scale bar is 50 µm); (**c**) EPED with an 8-µm-thick layer of Ag/AgCl ink deposited on top of the omniphobic paper (Ag/AgCl facing up). The inset shows an SEM image of the Ag/AgCl/paper electrode, demonstrating that neither the functionalization of the paper nor the subsequent deposition of Ag/AgCl ink clogged the porous structure of the paper substrate (scale bar is 100 µm); (**d**) Conforming of EPEDs to skin bending and buckling due to severe compression; (**e**) Left: Representative stress-strain curve of an unpatterned paper substrate. Inset shows the experimental set up used. Right: Stress-strain curve of an Ag/AgCl EPED showing how the razor patterning of the EPED improves its stretching when compared to unpatterned paper. Inset shows the mechanical characterization of a representative EPED sample.

We functionalized the paper substrates by spraying the organosilane solution at ambient conditions and letting it dry in a desiccator at 36 Torr for 20 min [6]. The open mesh serpentine layout of the EPEDs was designed using Adobe Illustrator CC (Adobe Systems Inc., San Jose, CA, USA) according to geometries previously reported in [10,11]. The minimum line width of the serpentine layout was kept at 200 µm (Figure 1b and Figure 2b), the minimum resolution of our programmable razor printer (Silhouette Cameo^TM^, Silhouette America Inc., Lindon, UT, USA), which uses a 100-μm-thick blade as the cutting tool. Prior to shaping the serpentine layout of the EPEDs, we attached adhesive copper tape (for copper-based EPEDs, Figure 1) or stencil printed Ag/AgCl ink (for Ag/AgCl-based EPEDs) on the functionalized paper. These functionalized paper substrates covered with a conductive layer (thickness of the composite ranging 70–190 μm) were then attached to a water-soluble tape (Aquasol Corp., North Tonawanda, NY, USA), which acted as the transfer layer to mount the EPEDs on skin (Figure 1c–f). Prior to the placement of EPEDs on skin, we sprayed medical glue (Medique products, Fort Myers, FL, USA) over the skin to maintain the conformal contact of the EPEDs on stretching. The transfer layer was then dissolved under a stream of running water (Figure 1e,f).

### 2.3. Physiological Signal Measurement with EPEDs

We recorded ECG, EMG, and EOG signals using a three-electrode configuration [7]. The physiological signals were amplified, filtered, and displayed using a commercial electrophysiological recorder (Backyard Brains, Ann Arbor, MI, USA) coupled to a portable open-source microcontroller (UNO, Arduino Inc.). The thickness of the medical glue layer deposited on the skin is <2 µm [9], minimally affecting the performance of the EPED while recording physiological signals.

We attached external cables (28 AWG) directly onto the conductive layer of the EPEDs (over the contact pad area) using a small amount of low melting point soldering paste (SMD291AX, Chip Quik Inc., Niagara Falls, NY, USA). To perform underwater experiments, an extra layer of medical glue was deposited over the skin to encapsulate the flat connection between the EPEDs and the cables. Any excess of medical glue sprayed on the skin accumulated along the lateral walls of the EPED, preventing those exposed areas of the conductive layer from short-circuiting while under water.

To compare the performance of EPEDs with conventional electrodes, we ran parallel experiments using EPEDs and commercially available foam electrodes (Medline Industries Inc., Northfield, IL, USA). We coated the surface of the foam electrodes in contact with the skin with a conductive electrode gel (SPECTRA^®^ 360, Parker Laboratories Inc., Fairfield, NJ, USA) to ensure a good electrical contact.

### 2.4. Characterization of Wirelessly Powered EPEDs

To wirelessly power functional components, such as LEDs, we attached a miniaturized half-wave rectifier fabricated using SMD components (Table S3) to the EPED antennas. We studied the wireless power transfer capabilities of EPEDs by performing a frequency-dependent characterization using a vector network analyzer (E5071B ENA, Agilent Technologies, Santa Clara, CA, USA). We used a copper coil (18 AWG wire, 6 turns, 5 cm diameter) connected to the network analyzer through an SMA connector (Digi-Key Electronics, Thief River Falls, MN, USA) to transfer wireless power to the EPEDs. All EPEDs were characterized passively at a distance of 15 cm from the center of the coil, in an orientation perpendicular to the axis of the coil. The network analyzer was programmed to record the real and imaginary parts of the impedance at 1601 frequency points linearly spaced in the range 1–20 MHz, finding the resonant frequency of the EPED using the min-phase method [39]. To enable the wireless powering of EPEDs, the coil was excited at the resonant frequency with a sinusoidal signal generated by a waveform generator (DG4062 Series, RIGOL Technologies Inc., Beaverton, OR, USA).

### 2.5. Heat Therapy Using EPEDs

We used EPEDs with copper and Ag/AgCl ink as the conductive layers to apply heat uniformly to the skin of the user. The thermal distribution created by the EPEDs was imaged using an infrared (IR) camera (FLIR E8, Wilsonville, OR, USA). We used a DC power supply (DP832A, RIGOL Technologies Inc., Beaverton, OR, USA) to generate heat through the resistive EPED, applying power levels below FCC guidelines (<2 W). To ensure the accuracy of the real-time monitoring of the temperature of the skin, we kept the distance between the IR camera and the EPED fixed at 20 cm during all the experiments.

### 2.6. Scanning Electron Microscopy (SEM)

We used a scanning electron microscope (Nova NanoSEM 200, FEI, Hillsboro, OR, USA) to examine the structure of the fabricated EPEDs. Before imaging, we used a sputter coater (208HR, Cressington, UK) to create a uniform conductive coating of ~10 nm platinum, using a D.C. current of 40 mA for 60 s. SEM images of the samples were captured at an electron accelerating potential of 5 kV, spot size 3, and working distance of 5 mm using an Everhart-Thornley detector (ETD).

### 2.7. Mechanical Characterization of EPEDs

We obtained stress-strain characteristics of bare paper substrates as well as fabricated EPEDs using a universal testing machine (MTS insight 10, MTS Systems Corp., Eden Prairie, MN, USA) equipped with a 100 N load cell (model 661.18.F01) according to ASTM D828-16 specifications. For the bare paper substrates, we fixed the gage length at 50 mm and applied a loading rate of 10 mm/min; while for the EPEDs, we had a gage length of 10 mm (comparable to the size of the device) and a loading rate of 5 mm/min.

## 3. Results and Discussion

### 3.1. Working Principle of EPEDs

Figure 2a shows the two layers of the EPEDs fabricated using razor printing (fabrication steps detailed in Figure 1): a conductive 20-µm-thick copper film in contact with the skin of the user and a silanized paper support (thickness ranging from 70 to 180 µm) that exhibits a static contact angle of 156°. The silane used to render paper omniphobic (both hydrophobic and oleophobic) prevents the EPEDs from being wetted by aqueous solutions and organic liquids with surface tension as low as 28 mN m^–1^ [6]. The covalent bonds generated between the alkyl trichlorosilanes and the cellulose fibers of the paper during the functionalization process are stable both in ambient conditions and under water for temperatures up to 150 °C [34]. Moreover, the chemical modification of the cellulose fibers of the paper do not affect its porosity (Figure 2c inset), preserving the gas permeability of the paper. The razor printing method used to fabricate the EPED enables the fabrication of flexible electrodes with a linewidth of 200 µm (Figure 2b) and a thickness of 78 µm when using Ag/AgCl ink as the conductive layer (70 µm is the thickness of the paper and 8 µm is the average thickness of the Ag/AgCl ink, see Figure 2c). The low thickness of the EPEDs fabricated using razor printing ensure their conformability to skin even when it wrinkles due to compression forces (Figure 2d) [15]. After dissolving the water-soluble transfer layer, 70-μm-thick EPEDs adhere to the skin solely by van der Waals and capillary forces, without requiring the spray-on medical glue. However, for a more robust adhesion of the EPEDs, we sprayed the skin with medical glue in all cases, regardless of the thickness of the paper used as a substrate. Since the thickness of the sprayed layer of glue is very small (<2 μm; [9]), its use does not adversely affect the functionality of the EPEDs or increase experimental noise in any significant way. The solvent of the medical glue sprayed on the skin of the user prior to placing the EPED does not affect the wetting properties of the paper substrate, which remains omniphobic after the medical glue dries. The omniphobic cellulose fibers provide a mechanical reinforcement to the thin film metals and conductive inks used in the conductive layer of the EPED, allowing them to withstand accidental stresses up to 2.5 MPa without tearing (Figure 2e). Despite the limited stretchability of unpatterned paper (~4%), the serpentine pattern used in the design of the EPED electrodes, enables these epidermal devices to endure stretching up to ~58% before failure. As a comparison, the maximum strain of human skin is ~30% [40].

### 3.2. Realtime Monitoring of Cardiac Activity

**Figure 3:**
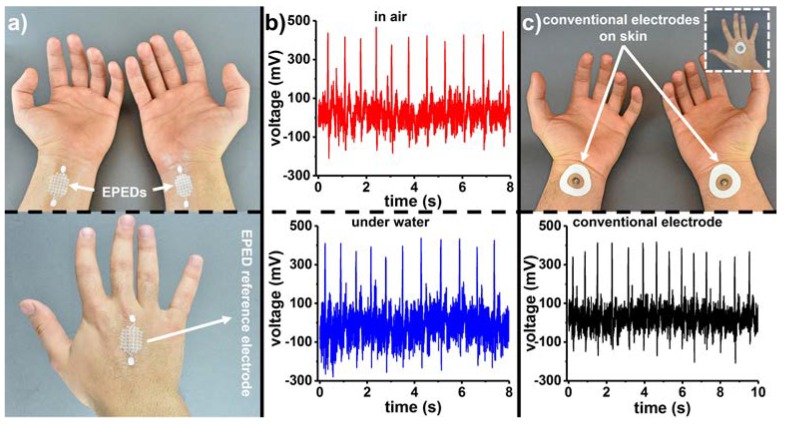
Comparison between the performance of razor printed copper-based EPEDs and conventional foam electrodes to record ECG signals: (**a**) Top: EPED measurement and ground electrodes used to record ECG signals from the wrist of a subject. Bottom: Reference EPED electrode; (**b**) Top: ECG signals recorded in air using razor printed EPEDs. Bottom: ECG signals recorded with both hands under water; (**c**) Top: Conventional foam electrodes placed at the same locations of the wrist as (**a**). The inset shows the location of the reference foam electrode on the back of the hand. Bottom: ECG signals recorded in air using conventional foam electrodes.

We recorded ECG signals from a human subject by attaching copper-based EPED electrodes on the wrist (measurement and ground) and the back of the hand (reference), as shown in Figure 3a. Figure 3b (top) shows the ECG signals recorded with the EPED electrodes. The silanization of the paper substrate to render EPEDs omniphobic allows us to capture high quality ECG signals even with the EPEDs completely immersed in water (Figure 3b bottom). We compared the signal to noise ratio (SNR) of EPED electrodes to conventional foam electrodes by placing them on the same locations of the hand (Figure 3c top). ECG measurements acquired by razor printed EPED electrodes (SNR_ECG-EPED,air_ = 12.20 dB, SNR_ECG-EPED,water_ = 10.37 dB; Table S2) exhibit no significant difference from conventional foam electrodes in air (Figure 3c bottom, SNR_ECG-foam,air_ = 11.28 dB). Conventional electrodes, though, cannot reliably capture ECG signals under water due to the swelling of their hydrogel terminals and their subsequent delamination and short-circuit.

### 3.3. Real-Time Monitoring of Muscle Activity

**Figure 4:**
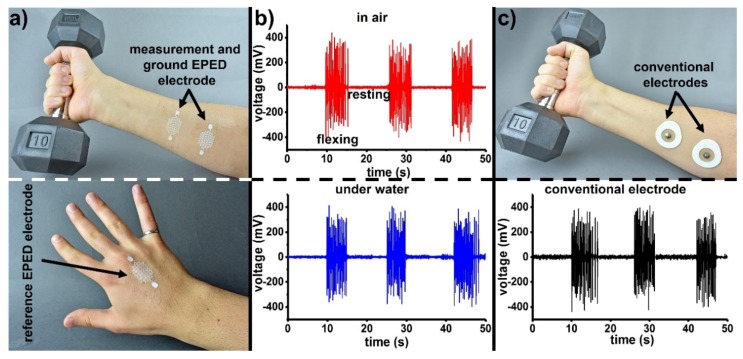
Comparison between the performance of razor printed EPEDs and conventional foam electrodes to monitor EMG signals: (**a**) Top: EPED measurement and ground electrodes used to record EMG signals from the forearm of an exercising subject. Bottom: Reference EPED electrode; (**b**) Top: EMG signals recorded in air using razor printed EPEDs. Bottom: EMG signals recorded under water; (**c**) Top: conventional foam electrodes placed at the same locations of the forearm as (**a**). Bottom: EMG signals recorded in air using conventional foam electrodes.

We used copper-based EPEDs to record EMG signals from the forearm by placing the measurement and ground electrodes along the flexor muscle and the reference electrode on the back of the hand (Figure 4a). Figure 4b (top) shows the EMG signals recorded with EPEDs while lifting a 4.5 kg dumbbell, holding it for 5 s, and resting for 10 s. The omniphobic character of the EPED provided by the silanization of the paper substrate, enables EMG signals to be captured in high moisture environments without significant experimental noise or short circuiting the measuring electrodes. To demonstrate the moisture-independent collection of EMG signals, we repeated the measurements while keeping the arm in a water bath. We observed no significant difference in performance between the razor printed EPEDs (SNR_EMG-EPED,air_ = 31.79 dB, SNR_EMG-EPED,water_ = 30.16 dB; Table S2) and conventional foam electrodes (SNR_EMG-foam,air_ = 26.58 dB) to record EMG signals in air. Conventional electrodes, however, are not capable to record EMG signals under water due to the short-circuit of their terminals.

### 3.4. Monitoring Eye Motion

**Figure 5:**
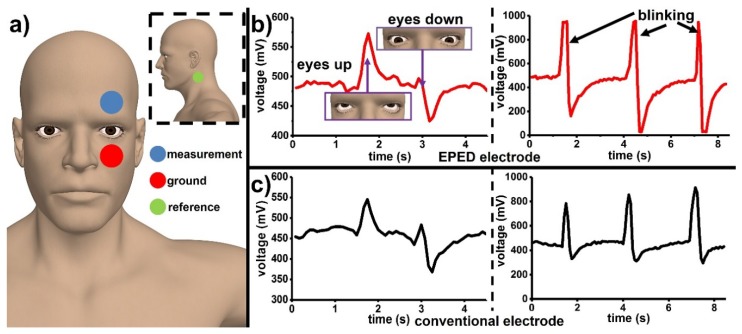
Monitoring eye motion using razor printed EPEDs: (**a**) Location of the measuring, ground, and reference copper-based EPED electrodes; (**b**) Left: EOG signal identifying eye movements (up and down) using EPEDs; Right: Identification of blinking events using EPEDs; (**c**) Left: Identification of eye movements (up and down) using conventional foam electrodes located as shown in (**a**); Right: Identification of blinking events using conventional foam electrodes.

We used three copper-based EPED electrodes placed on the cheekbone (ground), forehead (measurement), and neck (reference) to capture EOG signals and to monitor the movement of the eye (Figure 5a). The mechanical conformability of EPEDs enabled the identification of the movement of the eyes (up and down) as well as blinking events with minimal experimental noise (SNR_EOG-EPED,air_ = 33.77 dB; Figure 5b, Table S2). When compared with conventional foam electrodes (SNR_EOG-foam,air_ = 31.17 dB), EPEDs exhibit better performance upon the natural moves of the user (Figure 5c).

### 3.5. Wireless Powering of EPEDs

**Figure 6:**
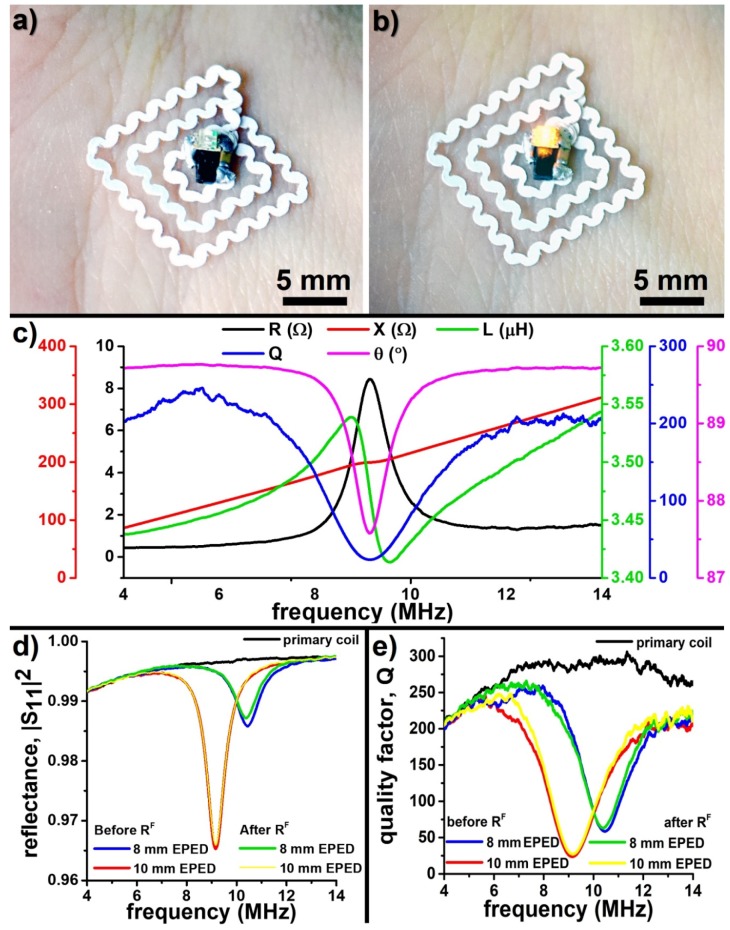
Electrical characteristics of wirelessly powered copper-based EPEDs: (**a**) Square EPED antenna (10 mm side) coupled with an SMD rectifier-LED circuit and attached to the skin of the wrist; (**b**) The LED is wirelessly powered using a primary coil 15 cm away (not shown in picture) running alternating currents at a resonant frequency of 9.0 MHz; (**c**) Frequency-dependent passive characteristics (Resistance, R, Reactance, X, Phase, θ, Inductance, L, and Quality factor, Q) of the EPED shown in (**a**,**b**). The frequency at which the phase θ is minimum is the resonant frequency of the EPED; (**d**,**e**) Effect of EPED size and silanization on the wireless power transfer efficiency (η = 1 − |S_11_|^2^) and the quality factor (Q). The silanization process has a negligible effect on η and Q. η and Q decrease when the size of the EPED is reduced.

The low resistivity (~20 nΩ m for copper-based EPEDs) of EPED antennas make them suitable for wireless power transfer based applications (Figure 6). Figure 6a,b shows a 10 mm EPED antenna with an LED and a rectifier circuit mounted on skin, being powered using far-field electromagnetic waves emitted from a primary coil placed 15 cm away. The geometry of the EPED antenna was chosen to match previously reported wireless epidermal stimulators [11]. This square pancake coil has only three loops to minimize their shaping with the razor printer, since we experimentally found that coils with three loops were able to efficiently power the LED wirelessly via inductive coupling at a distance of 15 cm. The line width of the antenna was made to match the minimum resolution of our razor printer (~200 µm). After the coil was shaped with the razor printer, we folded the external end of the coil towards the center to make both ends of the coil to rest flat at a distance of 2 mm and soldered the SMD components (Table S3) between the ends of the coil (Figure 6a,b). We analyzed the frequency-dependent electrical characteristics of this EPED using methods described in Section 2.4 (Figure 6c). We recorded the real and imaginary components of the impedance, Z = R + jX, |Z| = (R^2^ + X^2^)^1/2^, where the real part, R, is the resistance, and the imaginary part, X, is the reactance. We used the recorded components of the impedance as a function of frequency to calculate electrical characteristics of the EPEDs such as inductance L = X/2πf, phase θ = tan^−1^ (X/R), and quality factor Q = X/R. The resonant frequency f_0_ of the EPED is determined by the min-phase method [39,41]: the frequency at which the θ response is minimized is taken as the resonant frequency of the EPED when coupled with the primary coil. Since the primary coil is connected to the network analyzer for a one-port measurement, only the S_11_ parameter is recorded (Figure 6d). The wireless power transfer efficiency is calculated as η = (1 − |S_11_|^2^) × 100%, where |S_11_|^2^ is defined as the reflectance. We found that the omniphobic functionalization of the paper does not significantly modify the resonant frequency or the power transfer efficiency of the EPED (Figure 6d,e). Modifying the values of the capacitors used to rectify the wireless signal, the wireless power transfer efficiency of the EPED can be easily optimized for a given frequency following the impedance-matching optimization method for magnetic resonance coupling systems [42].

**Figure 7:**
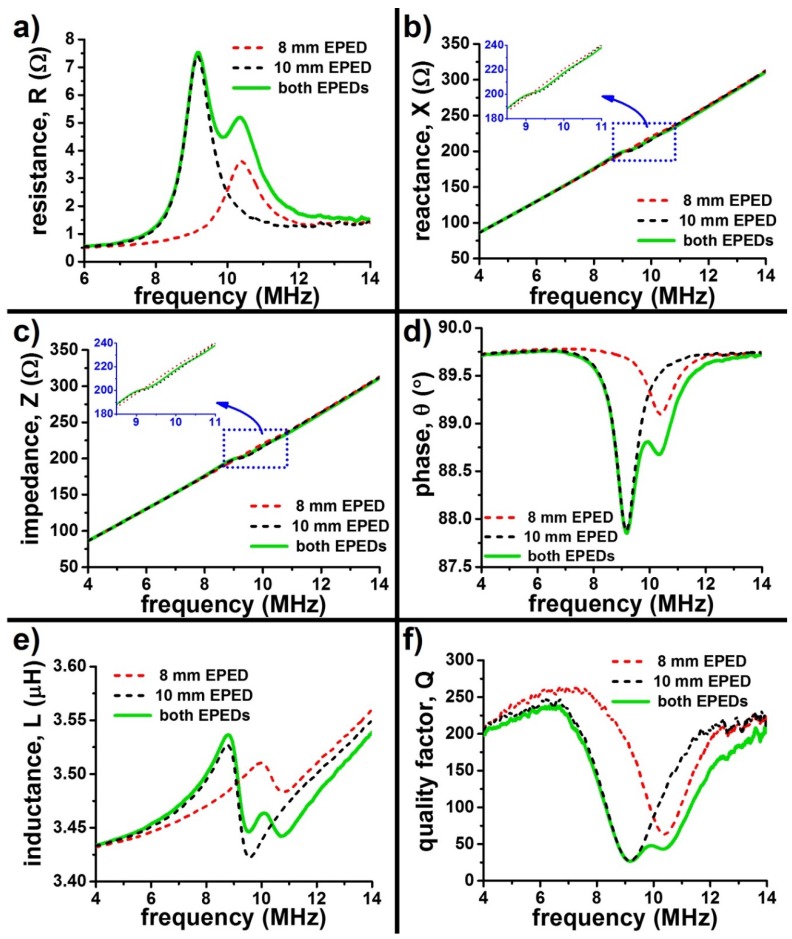
Selective powering of multiple EPEDs by varying excitation frequency, using the same primary coil. The green solid curves in all panels correspond to the frequency dependent passive characteristics when both EPEDs (8 mm and 10 mm side) are 15 cm away from the primary coil. The dashed lines represent individual characteristics: (**a**) Resistance, R; (**b**) Reactance, X; (**c**) Impedance, Z; (**d**) Phase, θ; (**e**) Inductance, L; (**f**) Quality factor, Q.

Multiple EPEDs placed in close proximity can be selectively powered, if these EPEDs have different resonant frequency peaks (due to their different geometry or rectifying circuit). Figure 7 summarizes the passive electrical characteristics of a system of 2 EPEDs, one 8 mm and another 10 mm, placed side by side, but not in contact with each other. The different resonant frequencies of the EPEDs (9.0 MHz for 8 mm side EPED; 10.4 MHz for 10 mm side EPED) enable their selective activation, independently or at the same time, depending on the desired application (Figure 7d).

### 3.6. Localized Heat Therapy

**Figure 8:**
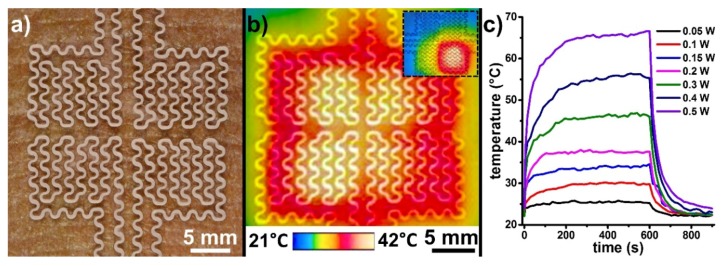
Application of localized heat therapy using razor printed EPEDs: (**a**) Copper-based thermotherapy EPED mounted on skin; (**b**) IR image of the EPED shown in (a) during thermotherapy (inset shows the application of localized heat by the selective activation of only one quadrant of the EPED); (**c**) Temperature-time response of the thermotherapy EPED for different D.C. powers.

Epidermal heat therapy is commonly employed in cancer treatments [43] and in orthopedics for alleviating joint pain [44]. Figure 8a shows a copper-based EPED fabricated to apply localized heat therapy on the skin. Heat is produced in accordance with Joule’s law of heating by running D.C. power through the EPED. The serpentine layout of this EPED was designed according to previously reported epidermal electronic devices [10,11] and razor printed with a minimum linewidth of 200 µm. We monitored the temperature distribution produced by the EPED using an IR camera, limiting the maximum temperature applied to the skin to 42 °C (Figure 8b). Each of the quadrants of the EPED has two independent contact pads that enable their individual activation to provide localized doses of heat (Figure 8b inset). Figure 8c shows the time-dependence of the heating process for different D.C. powers. The low specific heat of copper ensures that the EPED temperature rapidly stabilizes as power is applied, and restores quickly to room temperature once the power supply is turned off. The omniphobic properties of the EPED remain unaffected after the heating cycles.

### 3.7. Thermal Sensing

**Figure 9:**
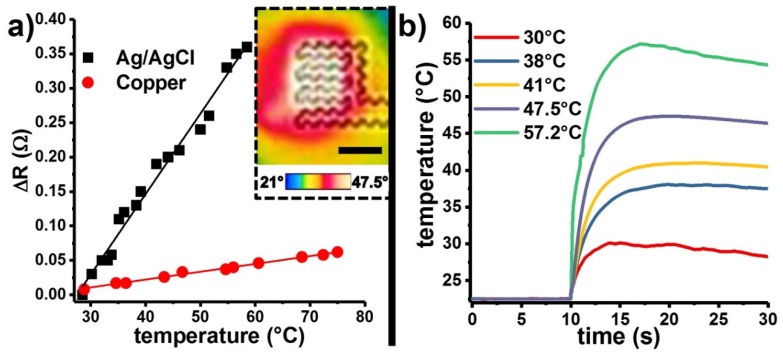
Sensing temperature using EPEDs: (**a**) Change of the EPED resistance as a function of temperature for EPEDs with a thin copper film (solid red dots) and Ag/AgCl ink (solid black squares) as conductive layers. The inset shows an IR image of an Ag/AgCl-based EPED when a small aluminum cylinder at 47.5 °C is placed on its surface for 10 s and then removed. Scale bar is 5 mm; (**b**) Response of the Ag/AgCl-based EPED thermometers when an aluminum cylinder at different temperatures is placed in contact with the EPED at t = 10 s.

The temperature dependence of the resistivity of the conductive layer of the EPEDs enables their use as wearable thermometers (Figure 9). The linear relationship between the increment in resistance of the EPED and its temperature allowed us to calculate the sensitivity of Ag/AgCl- and copper-based EPED thermometers (Figure 9a). The sensitivity of Ag/AgCl- and copper-based EPEDs are 0.01 Ω/°C and 0.001 Ω/°C, respectively. We characterized the time response of the Ag/AgCl EPEDs by placing them over a surface at room temperature (t = 0 s) and placing an aluminum cylinder preheated to different reference temperatures (t = 10 s). We observed that Ag/AgCl EPEDs required less than 1 s to reach reference temperatures in the clinically relevant range (Figure 9b).

## 4. Conclusions

This work reports the simple, inexpensive, and scalable, fabrication of epidermal paper-based electronic devices (EPEDs) using a bench-top razor printer. EPEDs fabricated using silanized paper can be used as moisture-insensitive epidermal electrodes, with a cost so low that it makes them compatible with single-use applications (see Table S1). Razor printed EPEDs fabricated using copper film or Ag/AgCl ink are easy to mount on skin, conforming to its natural moves, and exhibit good mechanical contact with the user and a stable electrical performance upon stretching. Copper-based EPEDs exhibit low resistivity values (~20 nΩ m), enabling their use as efficient electrophysiological monitors, thermotherapeutic devices, and wirelessly powered systems. The low resistance of copper-based EPEDs, however, makes it difficult to detect changes in the resistance caused by environmental temperature. Ag/AgCl-based EPEDs have higher resistivity values (~110 nΩ m), facilitating their use as temperature sensors since small changes in the environmental temperature induce larger changes in the resistance of the devices. The fibrous structure of the paper substrates of the EPEDs makes them breathable when their conductive layer is porous, such as Ag/AgCl-based EPEDs. The adhesion of a continuous copper film to the paper, however, compromises the passage of gases across the EPED. We demonstrated the omniphobic character of razor printed EPEDs by efficiently recording ECGs, EMGs, and EOGs in air and under water without any significant decrease in performance. The use of razor printing to fabricate EPEDs, at its present level of development, also has two limitations: (i) The minimum line width of the serpentine traces is 200 µm; (ii) The shear forces applied during high-speed cutting processes can lead to the delamination of the conductive layer from the omniphobic paper support if the adhesive used to secure both layers is not properly chosen. The wide range of adhesive materials and films compatible with razor printing, however, can ameliorate this limitation. We expect that the proposed method to fabricate inexpensive wearable electrodes will facilitate the adoption of epidermal electronics in personalized medicine, especially in resource-limited and home environments.

## Supplementary Materials

The following are available online at http://www.mdpi.com/2072-666X/9/9/420/s1, Table S1: Itemized cost per device of each of the components integrating a razor printed EPED; Table S2: Signal-to-Noise Ratio (SNR) for electrophysiological signals recorded with EPEDs and conventional foam electrodes; Table S3: Layout of half-wave rectifying circuit connected to EPED antenna.
