# Supplementary material for: Rapid Fabrication of Epidermal Paper-Based Electronic Devices Using Razor Printing
Source: Micromachines (Basel). 2018 Aug 22;9(9):420. doi: 10.3390/mi9090420 (PMC6187327; doi:10.3390/mi9090420)
Supplement: Supplementary file 1 [file micromachines-09-00420-s001.pdf]

Supporting information

Rapid Fabrication of Epidermal Paper-Based Electronic Devices using Razor Printing

Behnam Sadri [1], Debkalpa Goswami [1] and Ramses V. Martinez [1,2,*]

1: School of Industrial Engineering, Purdue University, West Lafayette, IN 47907, USA
2: Weldon School of Biomedical Engineering, Purdue University, West Lafayette, IN 47907, USA

Keywords: epidermal sensors; stretchable electronics; wireless power; hydrophobic paper; wearable stimulators; paper electronics; low-cost manufacture.

* Corresponding Author: rmartinez@purdue.edu, Tel.: +1-765-496-0399

1. Cost of fabrication

We estimate the cost of fabricating each of the razor printed EPEDs, without considering labor or capital expenses to be less than $0.07. This total cost, itemized in Table S1, is based on costs of small quantities of material and reagents and could be subject to lower prices on volume discounts.

Table S1. Itemized cost per device of each of the components integrating a razor printed EPED.

**Cost of razor printed EPED**	
Copper tape	$ 0.0217
Whatman #1 paper	$ 0.005
Silver ink	$ 0.015
Water soluble tape	$ 0.019
Omniphobic functionalization	$ 0.006
**Total cost**	**$ 0.0667**

2. Signal-to-Noise Ratios (SNR) of measured physiological signals

We calculated the SNR values for EPEDs (in air and under water) and conventional foam electrodes using the following formula:

$$SNR = 10\log_{10}\frac{A_{signal}^2}{A_{noise}^2}$$

Where A_{signal} and A_{noise} are the amplitudes of the signal and the noise, respectively.

Table S2. Signal-to-Noise Ratio (SNR) for electrophysiological signals recorded with EPEDs and conventional foam electrodes.

Physiological Signal	EPED (in air)	EPED (under water)	Foam Electrode (in air)
ECG	12.20 dB	10.37 dB	11.28 dB
EMG	31.79 dB	30.16 dB	26.58 dB
EOG	33.77 dB	—	31.17 dB

3. Components of the wirelessly powered EPED antenna

Table S3. Layout of half-wave rectifying circuit connected to EPED antenna.

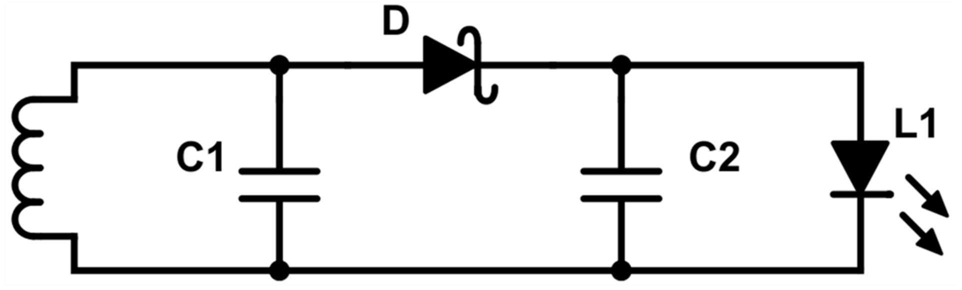

**SMD Components**
C1 = 4.7 nF
C2 = 220 nF
D = HSMS-282x
L1 = SML-211DTT86
